# Naringenin Accelerates Diabetic Wound Healing via Regulating Macrophage M2 Polarization and Efferocytosis

**DOI:** 10.1002/fsn3.70688

**Published:** 2025-08-01

**Authors:** Beizhi Wang, Yumeng Huang, Youjun Ding, Jingyi Chen, Yutong Chen, Hao Zhang, Qian Tan

**Affiliations:** ^1^ Department of Burns and Plastic Surgery, Nanjing Drum Tower Hospital, Clinical College Nanjing University of Chinese Medicine Nanjing China; ^2^ Department of Burns and Plastic Surgery, Nanjing Drum Tower Hospital, Clinical College Jiangsu University Nanjing China; ^3^ Department of Emergency Surgery The Fourth Affiliated Hospital of Jiangsu University (Zhenjiang Fourth People's Hospital) Zhenjiang China; ^4^ Department of Burns and Plastic Surgery, Nanjing Drum Tower Hospital, Affiliated Hospital of Medical School Nanjing University Nanjing China

**Keywords:** diabetes, macrophage, naringenin, wound healing

## Abstract

Diabetic wounds that do not heal are a significant complication of diabetes, among which chronic inflammation serves as a critical contributing factor. The regulation of inflammation in diabetic wound healing is significantly impacted by macrophages via their efferocytosis and polarization activities. Naringenin is a natural flavonoid that has been recognized for its anti‐inflammatory effects and potential therapeutic benefits in treating diabetes‐related conditions. This research examines how naringenin impacts non‐healing diabetic wounds and delves into the mechanisms behind it. The study involved male C57BL/6 mice categorized into three groups: control, diabetes, and diabetes with naringenin treatment. Researchers assessed the effect of naringenin on wound healing by applying it topically to a diabetic mouse model induced with streptozotocin (STZ). Moreover, researchers used THP‐1 cells in vitro to examine the effects of naringenin on the polarization of macrophages to the M2 phenotype and their efferocytosis. Subsequently, the effects of naringenin treatment were assessed using RT‐qPCR, western blot, immunofluorescence, and additional assays. Animal experiments demonstrated that naringenin significantly accelerated diabetic wound healing. Naringenin decreased inflammatory cytokines, promoted M2 macrophage polarization, and enhanced macrophage efferocytosis in wound healing. Consistent with animal studies, naringenin inhibits macrophage M1 polarization while augmenting M2 polarization in THP‐1 cells, with ML385 specifically rescuing the M1 suppression. In addition, naringenin also promoted macrophage efferocytosis in a THP‐1/Jurkat apoptotic co‐culture mode. Naringenin significantly promotes diabetic wound healing via ameliorating wound inflammation and exerts its therapeutic effects through promoting M2 macrophages and enhancing efferocytosis.

## Graphical abstract

Naringenin is a natural flavonoid that has been recognized for its anti‐inflammatory effects and potential therapeutic benefits in treating diabetes‐related conditions. This research investigated that naringenin significantly promotes diabetic wound healing via ameliorating wound inflammation and exerts its therapeutic effects through promoting M2 macrophages and enhancing efferocytosis.

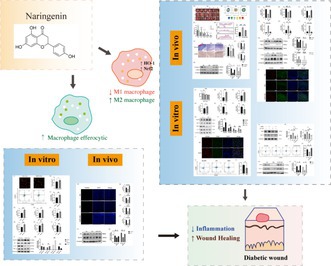

**Abbreviations**
ANOVA: analysis of variance
Arg‐1: arginase 1
BSA: bovine serum albumin
CCK8: cell counting kit‐8
CD: cluster of differentiation
DAB: diaminobenzidine
DAPI: 4,6‐diamidino‐2‐phenylindole
DM: diabetes group
DMSO: dimethyl sulfoxide
F4/80: mouse EGF‐like module‐containing mucin‐like hormone receptor‐like 1
FBS: foetal bovine serum
HE: hematoxylin–eosin
HRP: horseradish peroxidase
IF: Immunofluorescence
IL: Interleukin
iNOS: Inducible nitric oxide synthase
LPS: lipopolysaccharide
MT: Masson's trichrome
NAR: diabetes + naringenin group
NC: control group
PBS: phosphate buffer saline
PE: P‐phycoerythrin
PMA: phorbol 12‐myristate 13‐acetate
PVDF: polyvinylidene fluoride
ROS: reactive oxygen species
RT‐qPCR: real‐time quantitative PCR
STZ: streptozotocin
TBST: tris buffered saline tween.
TGF‐β: transforming growth factor‐β
TNF‐α: tumor necrosis factor‐α
WB: western blot
α‐SMA: alpha‐smooth muscle actin

## 1 Introduction

The global health burden of diabetes mellitus is considerable, currently impacting 463 million individuals, and is expected to grow to 578 million cases by 2030 according to epidemiological forecasts (Saeedi et al. 2019). Diabetic wounds are a severe complication of diabetes that can lead to high morbidity, recurrence, mortality, and many non‐traumatic limb amputations (Armstrong et al. 2017; Falanga 2005). Effective and convenient treatments for these wounds are urgently needed.

Normal wounds healing encompasses a series of complex and dynamically overlapping stages: namely hemostasis, inflammation, proliferation, and remodeling (Baum and Arpey 2005). Unlike normal wound healing, diabetic wounds are affected by pathological processes such as prolonged inflammation, oxidative stress, and apoptosis (Arya et al. 2014). The persistent inflammation in diabetic wounds is primarily a result of the inflammatory factors secreted by neutrophils and macrophages, which subsequently inflict additional damage on the surrounding healthy tissues and cells (Zhao et al. 2016).

In diabetic wound healing, macrophages are key players and are distinguished as either classically activated (M1) or alternatively activated (M2). During the initial inflammatory phase, M1 macrophages act as the principal source of inflammatory mediators, which facilitate the additional pro‐inflammatory macrophages in the wound area (Rohm et al. 2022). Macrophages undergo a shift from M1 to M2 at the end of the inflammatory phase, secreting cytokines including IL‐4 and IL‐10 (Morey et al. 2019). This transition aids the shift from inflammation to proliferation. However, an imbalance in the M1/M2 ratio in diabetic wounds frequently leads to sustained inflammation, thereby hindering and extending the healing process (Barman and Koh 2020). Additionally, wound healing requires macrophages to clear apoptotic cells (a process called efferocytosis) to eliminate inflammation (Boada‐Romero et al. 2020; Malissen et al. 2014). Efferocytosis is mediated primarily by M2 macrophages (Ariel and Serhan 2012; Bystrom et al. 2008). Insufficient efferocytosis, a common complication in diabetes, leads to persistent inflammation due to the accumulation of apoptotic cells, reduced growth factors, and impaired granulation tissue formation, ultimately hindering the healing of diabetic wounds (Ge et al. 2022). As previously discussed, promoting M2 macrophage polarization and facilitating macrophage efferocytosis can mitigate excessive inflammatory responses and accelerate diabetic wound healing.

Naringenin (4,5,7‐trihydroxyflavanone) is a naturally occurring flavonoid derived from citrus species and tomatoes that exhibits notable anti‐inflammatory and antioxidant properties (Renugadevi and Prabu 2009; Shi et al. 2009) Multiple experimental studies have substantiated that naringenin promotes M2 macrophage polarization (Chaen et al. 2019; Chen et al. 2022; Jia et al. 2022; Ma, Liu, et al. 2023). Furthermore, naringenin demonstrates potential in mitigating diabetic nephropathy and improving endothelial dysfunction induced by a high glucose environment (Zhang et al. 2017). Based on these studies, we hypothesized that naringenin facilitates diabetic wound healing. As we all know, the Nrf2/HO‐1 signaling pathway is crucial for reducing inflammation and combating oxidative stress (Cuadrado et al. 2018; Vallion and Kerdine‐Römer 2022). Additionally, the Nrf2/HO‐1 pathway is crucial in regulating macrophage phenotypes (Lv et al. 2021; Sha et al. 2023). Activation of Nrf2‐mediated antioxidant gene expression can attenuate M1 macrophage activity and decrease the generation of reactive oxygen species (ROS) (Kobayashi et al. 2016). Besides, research demonstrated that naringenin confers protection against acute pancreatitis and inflammatory pain through the Nrf2/HO‐1 pathways (Li et al. 2018; Manchope et al. 2016). However, the underlying mechanisms of naringenin's impact on macrophage polarization have not yet been elucidated.

To investigate the effects of naringenin on diabetic wound healing, we employed a model of diabetic mice with full‐thickness skin wounds induced by STZ. Additionally, this study aims to perform a series of experiments to elucidate the effect of naringenin in reducing inflammation through M2 polarization and efferocytosis, thereby enhancing diabetic wound healing. Our findings demonstrate the potential of naringenin in improving macrophage function for diabetic wound healing.

## 2 Material and Methods

### 2.1 Antibodies and Reagents

Naringenin (N5893) was obtained from Sigma‐Aldrich with a purity ≥ 95%. The phorbol‐12‐myristate‐13‐acetate (PMA; P8139), glucose (D9434), streptozotocin (STZ; S0130) and lipopolysaccharide (LPS; L2654) were also obtained from Sigma‐Aldrich. Dimethyl sulfoxide (DMSO; HY‐Y0320) was obtained from MCE. The PE anti‐human CD86 (305405) antibody was bought from BioLegend. The Alexa Fluor 647 Mouse Anti‐Human CD68 (Y1/82A) was bought from BD Pharmingen. The primary antibodies against Collagen I (72026) were purchased from Cell Signal Technology. α‐SMA (ET1607‐43), Arg‐1 (ET1605‐8), CD206 (ET1702‐04), MerTK (ET1602‐21), Axl (R1406‐3), and Nrf2 (HA721432) were provided by Huabio. iNOS (22226‐1‐AP), IL‐1β (16806‐1‐AP), MerTK (27900‐1‐AP), and β‐actin (20536‐1‐AP) were obtained from Proteintech. IL‐1β (ab216995), TNF‐α (ab255275), and HO‐1 (ab52947) were purchased from Abcam. The Nrf2/HO‐1 pathway inhibitor, ML385 (T4360), was bought from TargetMol.

### 2.2 Animal Experiments

Male C57BL/6 mice, aged 6 to 8 weeks and weighing 20 to 25 g, were obtained from Jiangsu Jicui Yaokang Biotechnology. The mice were given an adaptive feeding plan for 1 week before the experiment and were kept on a 12‐h light/dark cycle. Subsequently, the mice were allocated to three groups randomly: control group (NC), diabetes group (DM), and diabetes + naringenin group (NAR + DM), with 16 mice in each group. All mice were treated as independent biological replicates. Mice were allocated to different experimental batch groups and performed at different times to control for batch effects. A diabetic mouse model was established via intraperitoneal injection of streptozotocin (STZ) dissolved in sodium citrate buffer (0.1 M, pH 4.5). Mice received daily injections at a dosage of 50 mg/kg (based on the body weight of the mice) for 5 days. Fasting blood glucose levels were measured 4 weeks after the final STZ injection. Mice with fasting blood glucose (FBG) levels ≥ 16.7 mmol/L were classified as diabetic, a criterion aligned with standard protocols in diabetic rodent models, as supported by prior studies (Park et al. 2024; Wang et al. 2024). FBG measurements were repeated prior to and during the intervention to confirm consistent hyperglycemia.

The mice were positioned prone and anesthetized with isoflurane. A sterile skin punch biopsy was used to create 10 mm full‐thickness wounds on the dorsal skin of mice to study the healing differences between diabetic and non‐diabetic wounds (Ding et al. 2022; Li et al. 2022; Ma, Ding, et al. 2023). A silicone splint was applied using 5–0 nylon to inhibit skin contraction. Naringenin was first dissolved in DMSO to make a stock solution with a concentration of 100 mM (−20°C, dark storage). Fresh dilutions to 20 μmol/L were made with PBS before subcutaneous injection and kept on ice to ensure stability. The NAR + DM group received a 100 μL naringenin solution (20 μmol/L) through intradermal injection around each wound site to study its effect on diabetic wound healing (Sun et al. 2023). Control groups (NC and DM) received equivalent volumes of PBS under identical conditions. The wound healing process was photographed at various times and analyzed with ImageJ software. The edge tissues of the wound were collected for further analysis.

### 2.3 H&E and MT Staining

After being fixed overnight in a 4% paraformaldehyde solution, the skin specimens were dehydrated and embedded in paraffin. Histological examination was performed on 5 μm thick skin paraffin sections. Finally, these sections were stained with either hematoxylin and eosin (H&E) or Masson's trichrome (MT). The histological sections were used to observe and monitor the morphological structure within the skin.

### 2.4 Immunohistochemistry

The process began with deparaffinization and hydration of paraffin‐embedded sections, followed by quenching endogenous peroxidase activity using a 3% H_2_O_2_ solution for a duration of 10 min in preparation for antigen restoration. Subsequently, the sections were subjected to a blocking process with 2% bovine serum albumin (BSA) at room temperature for an hour, then incubated overnight with the primary antibody α‐SMA. The sections were then exposed to an incubation period with horseradish peroxidase (HRP)‐labeled secondary antibodies for an hour, following which a DAB substrate solution was employed as the chromogen. The final stage of the process involved capturing images using a THUNDER Imager microscope, manufactured by Leica, Germany.

### 2.5 Immunofluorescence Analysis and TUNEL Staining

The paraffin‐embedded sections underwent a dewaxing process for rehydration, followed by antigen repair solution for repair. Subsequently, these sections were rinsed 3 times with PBS and blocked with a 3% BSA solution for 1 h at 37°C. To analyze M2 phenotype macrophages, the sections were incubated overnight with F4/80 and CD206 antibodies at 4°C. The sections were washed three times with PBS‐0.1% Tween‐20 solution after incubation and then incubated with secondary antibodies for an hour at room temperature, ensuring they were kept away from light. The sections underwent a final step of DAPI counterstaining for 5 min before being observed with immunofluorescence microscopy.

Apoptotic cells were identified using a one‐step TUNEL assay kit (Pricella, China) according to the manufacturer's guidelines. TUNEL‐positive apoptotic cells in the wound area were quantified by fluorescence microscopy.

### 2.6 Cell Culture and Treatment Procedures

THP‐1 and Jurkat human cells, obtained from the Cell Bank of the Chinese Academy of Sciences, were grown in RPMI1640 medium (Keygene Biotech, China) supplemented with 1% Penicillin–Streptomycin–Amphotericin B Solution and 10% fetal bovine serum (Vazyme, China). THP‐1 cells were differentiated with 100 nmol/L PMA for 24 h, then treated with 100 ng/mL LPS for 48 h. Subsequently, naringenin (20 μmol/L) or an equal amount of PBS was added, and incubation continued for another 24 h. In addition, ML385 (5 μmol/L), a specific Nrf2/HO‐1 pathway inhibitor, was administered 24 h prior to naringenin treatment.

### 2.7 CCK‐8 Cell Viability Assay

The experiment examined the proliferation of THP‐1 cells in response to various concentrations of naringenin, utilizing a CCK8 assay kit (Beyotime Biotech, China). In a 96‐well plate, THP‐1 cells were inoculated at 3 × 10^4^ cells per well, subsequently treated with naringenin at concentrations of 0, 20, 40, 60, 80, and 100 μmol/L for a duration of 24 h, with a control group set at 0 μmol/L. The procedure involved adding 10 μL of the Cell Counting Kit‐8 solution to each well at predetermined intervals. Absorbance at 450 nm was measured with a microplate reader (Thermo, USA) after an incubation of approximately 2 h. Calculate cell viability as the absorbance ratio between the treatment and control wells.

### 2.8 Preparation of Apoptotic Cells

Jurkat cells are beneficial for efferocytosis assays due to their fast growth and reliable induction of apoptosis (Maschalidi et al. 2022; Schappe et al. 2022). We added the Jurkat cells suspension to the culture dish, exposed it to 254‐nm ultraviolet light for 15 min (Fadok et al. 1998), and then incubated for 3 h at 37°C with 5% CO_2_. The apoptotic cells were centrifuged and resuspended in RPMI1640 culture medium after 3 h. Prior to their application in efferocytosis assays, apoptotic Jurkat cells were tagged with 1 mM CMFDA (Yeasen, China) for a duration of 30 min.

### 2.9 Efferocytosis Assay

An efferocytosis assay was conducted largely following the method described in the reference (Kourtzelis et al. 2019). In short, THP‐1 cells were maintained in 6‐well plates with a density of 1 × 10^6^ cells/mL. Once adhered, they were stained with DiI (Yeasen, China) for 20 min. Five‐fold CMFDA‐labeled apoptotic Jurkat cells were then added to the wells. After 4 h of co‐culture, the unengulfed and suspended apoptotic cells were removed by washing with PBS three times. Efferocytosis was assessed by counting cells that engulfed green fluorescent apoptotic bodies (Suresh Babu et al. 2016). We observed the ability of THP‐1 macrophages to clear apoptotic cells under a fluorescence microscope. For flow cytometry, THP‐1 cells were labeled with APC‐CD68 while apoptotic Jurkat cells were labeled with CMFDA. The remaining steps are similar to those described above. The proportion of phagocytic macrophages was calculated by dividing the CMFDA cell population by the total CD68+ cell count and multiplying by 100.

### 2.10 RNA Purification and RT‐qPCR Analysis

RNA from tissues or cells was isolated utilizing TRIzol. The HiScript III‐RT kit (Vazyme, China) was employed for the reverse transcription of RNA. The qPCR reactions utilized ChamQ Universal SYBR qPCR Master Mix (Vazyme, China). Following the normalization of relative expression ratios to β‐actin, the target gene's relative expression was computed using the 2^−ΔΔ*CT*^ method. Table 1 lists the primer sequences.

**TABLE 1:** List of primer sequence sets used for RT‐qPCR.

Primer name	Forward primers	Reverse primers
Mouse‐COLa	ATGTGCCACTCTGACTGGAA	TCCATCGGTCATGCTCTCTC
Mouse‐α‐SMA	GCTATTCAGGCTGTGCTGTC	GGTAGTCGGTGAGATCTCGG
Mouse‐Axl	TGCACAAGATCAGAGCTGGA	CGGCCATGAACTTCACTAGC
Mouse‐MerTK	TCCTTTTGCTGCAGTCACAC	TCAGCTGGCTTCACATCAGA
Mouse‐Tryo3	TGAAGGATGGGGAGGAAACC	AAAACAGAGGGAGAGGGAGC
Mouse‐LRP1	AACCTTATGAATCCACGCGC	TTCTTGGGGCCATCATCAGT
Mouse‐IL‐6	CTGTGAAGTCTCCTCTCCGG	CTGTGAAGTCTCCTCTCCGG
Mouse‐IL‐1β	ACTCATTGTGGCTGTGGAGA	TTGTTCATCTCGGAGCCTGT
Mouse‐iNOS	CCCCGCTACTACTCCATCAG	CCACTGACACTTCGCACAAA
Mouse‐Arg‐1	CTGAGCTTTGATGTCGACGG	TCCTCTGCTGTCTTCCCAAG
Mouse‐CD206	TGGATGGATGGGAGCAAAGT	GCTGCTGTTATGTCTCTGGC
Mouse‐IL‐10	GGTGAGAAGCTGAAGACCCT	TGTCTAGGTCCTGGAGTCCA
Mouse‐β‐Actin	CCTCTATGCCAACACAGTGC	CCTGCTTGCTGATCCACATC
Human‐LRP1	CTGACTGGCGAACAAACACA	ACGGTCCGGTTGTAGTTGAT
Human‐Tryo3	GCCCCTTTCCAACTGTCTTG	GCAGTGCTTGAAGGTGAACA
Human‐Axl	GAGGGAGAGTTTGGAGCTGT	GAAACAGACACCGATGAGCC
Human‐MerTK	TCTGTCGAATCAAAGCCCCT	AACGCTGCACACTGGTTATG
Human‐Arg‐1	GTGGAAGAAGGCCCTACAGT	GCTTTTCCCACAGACCTTGG
Human‐CD206	AACGGACTGGGTTGCTATCA	CCCGATCCCTTGTAGAGCAT
Human‐TGF‐β	TTGAGACTTTTCCGTTGCCG	CGAGGTCTGGGGAAAAGTCT
Human‐TNF‐α	AGGACCAGCTAAGAGGGAGA	CCCGGATCATGCTTTCAGTG
Human‐IL‐6	AGTCCTGATCCAGTTCCTGC	CTACATTTGCCGAAGAGCCC
Human‐IL‐10	GTTCTTTGGGGAGCCAACAG	GCTCCCTGGTTTCTCTTCCT
Human‐β‐Actin	CATCCGCAAAGACCTGTACG	CCTGCTTGCTGATCCACATC

### 2.11 Western Blotting

The extraction of protein was carried out with RIPA Lysis (Beyotime Biotech, China) and the concentrations were assessed using a BCA protein assay kit (Beyotime Biotech, China). After separation on 10% SDS‐PAGE gels, the protein samples were transferred to PVDF membranes (Bio‐Rad, USA) and blocked with 5% skimmed milk in TBS‐T for 90 min. The membranes that were blocked were exposed to primary antibodies overnight at 4°C, followed by treatment with secondary antibodies for 1 h at room temperature. They were then incubated with ECL hypersensitive luminescent solution (Vazyme, China) and imaged using a Tanon 6200 Luminescent Imaging Workstation (Tanon, China).

### 2.12 Flow Cytometry

The proportion of M1 type macrophages was determined by incubating cells with APC‐CD68 and PE‐CD86 antibodies, followed by staining for 30 min in darkness at 4°C. A BD AccuriC6 Plus cytometer was used to process and analyze all samples, and FlowJo v10.0 software was employed for data analysis.

### 2.13 Statistical Analysis

Each independent experiment was replicated a minimum of three times. All data were statistically analyzed utilizing GraphPad Prism 10.0 software. Group statistical analyses were compared employing the t‐test for two groups or one‐way ANOVA for three groups. The data are shown as the mean ± standard error of the mean (SEM), and a *p*‐value under 0.05 signifies statistical significance. Tukey's HSD post hoc test was applied for multiple comparison correction following ANOVA.

## 3 Results

### 3.1 Topical Administration Naringenin Improves Wound Healing in Diabetic Mice

**FIGURE 1:**
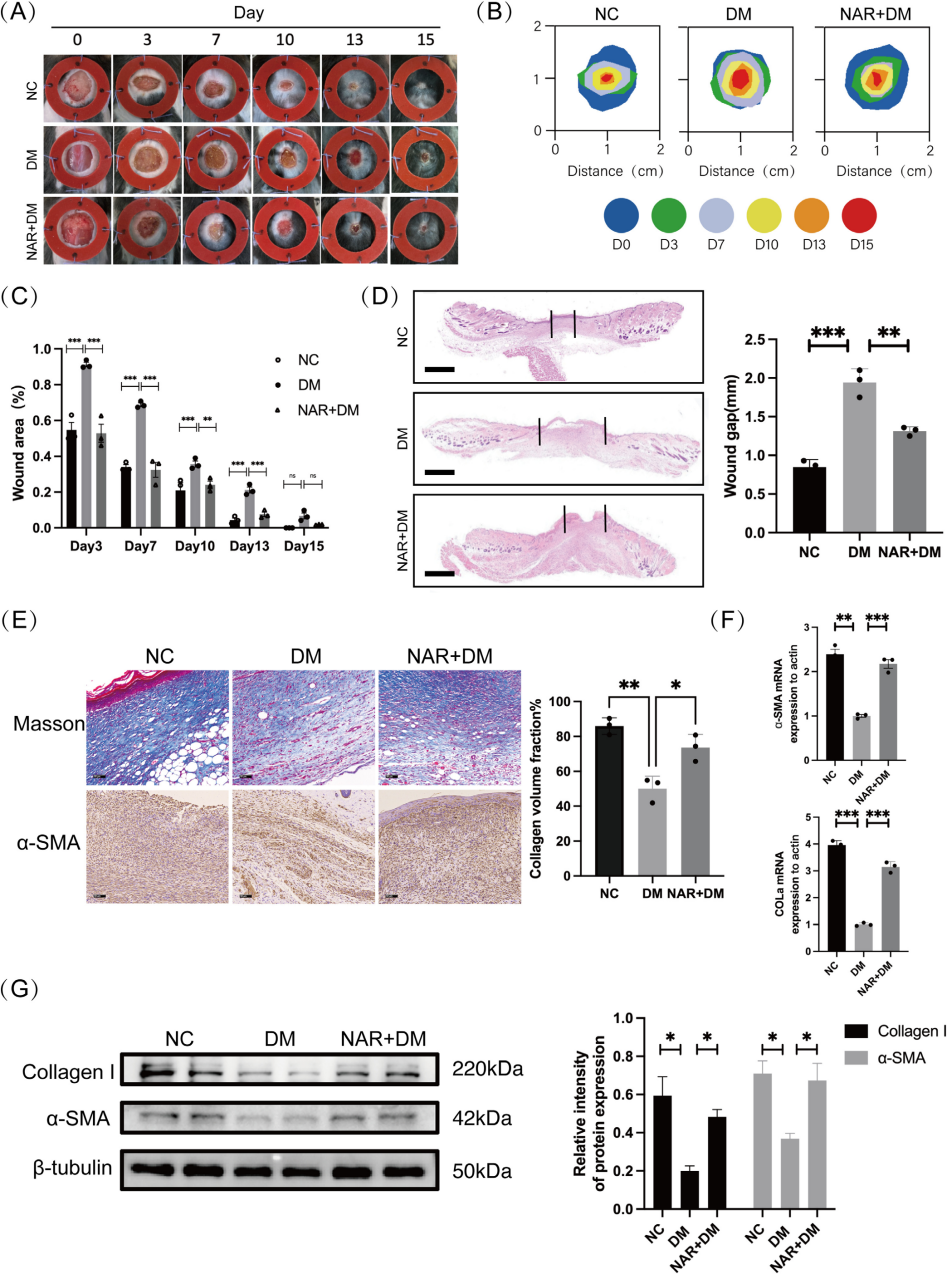
Treatment with naringenin accelerates wound healing in diabetic mice. (A) Photos of wound healing on Days 0, 3, 7, 10, 13, and 15 in various groups. (B) Simulation plots of wound healing in each group for 15 days. (C) Wound area (%) of different groups at Days 3, 7, 10, 13, and 15. (D) H&E staining showed wound gaps across various groups on Day 12 (Scale bar, 1 mm). (E) MT staining and immunohistochemistry images of α‐SMA in different groups on Day 12 (Scale bar, 50 μm). (F) qPCR and (G) Western blot were performed to identify the expression of Collagen I and α‐SMA. β‐tubulin served as the internal reference. Data are shown as means ± SEMs (*n* = 3), with significance levels marked as *p < 0.05;**p < 0.01; ***p < 0.001. NC: group without treatment; DM: group with diabetes; NAR + DM: group with diabetes treated with naringenin.

To assess the influence of naringenin on diabetic wounds, 10‐mm full‐thickness dorsal skin wounds were made on both normal and diabetic mice. After 15 days, wound healing in the DM group was slower than in the NC group, and the application of naringenin accelerated wound healing relative to the DM group. Significantly, the wound area in the DM group was much larger compared to the NC and NAR + DM groups on Days 3, 7, 10, and 13 (Figure 1A–C). Additionally, H&E staining indicated that the wound gaps in the DM group were significantly larger (Figure 1D). To further assess the quality of healing and collagen deposition, we performed α‐SMA immunohistochemical staining and MT staining. The results demonstrated that the DM group exhibited disorganization and loss of the collagen network, whereas the NAR + DM group markedly enhanced collagen deposition (Figure 1E). We proceeded to analyze the levels of α‐SMA and Collagen I, markers of muscle fibroblasts, in wound tissues through quantitative PCR (qPCR) and western blot (WB) analyses (Figure 1F,G). The DM group showed much lower levels of α‐SMA and Collagen I than the NC group; however, these levels significantly rose after naringenin treatment. These observations show that the DM group experienced a significantly slower wound healing process than the NC group, while naringenin treatment greatly enhanced healing in diabetic mice.

### 3.2 Naringenin Promotes M2 Macrophage Polarization and Reduces Inflammation in the Wound of Diabetic Mice

**FIGURE 2:**
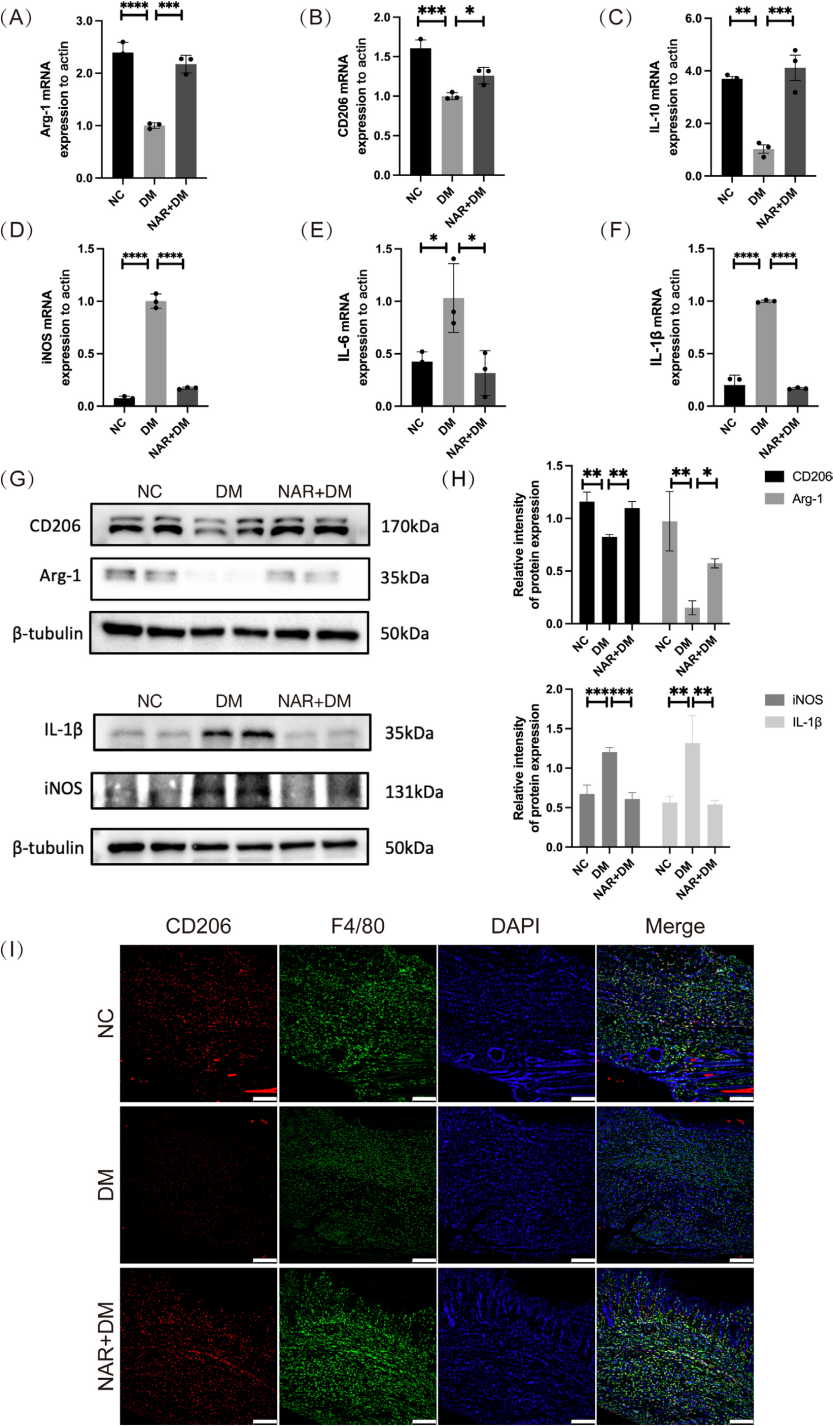
Naringenin enhances M2 macrophages polarization and suppresses inflammation in wound tissues. (A–F) mRNA expression levels of Arg‐1, CD206, IL‐10, iNOS, IL‐6, IL‐1β were detected by qPCR in wound tissues. (G, H) Quantitative analysis of the expression of iNOS, IL‐1β, Arg‐1, CD206 by western blot in different groups. β‐tubulin served as an internal reference. (I) Representative IF staining displayed CD206+ (red), F4/80+ (green) cells in different groups, with blue fluorescence indicating cell nucleus (scale bar, 200 μm). Data are shown as means ± SEMs (*n* = 3), with significance levels marked as *p < 0.05;**p < 0.01; ***p < 0.001. NC: group without treatment; DM: group with diabetes; NAR + DM: group with diabetes treated with naringenin.

We employed qPCR to examine inflammatory factors and markers of M2 macrophages to determine if naringenin could enhance M2 macrophage polarization in diabetic wounds. Compared to the NC group, the DM group exhibited notably lower levels of M2 macrophage markers (Arg‐1, CD206, IL‐10), but this trend was reversed with naringenin treatment (Figure 2A–C). The DM group showed increased expression of inflammatory factors (iNOS, IL‐6, IL‐1β) compared to the NC group, whereas naringenin treatment effectively lowered these levels (Figure 2D–F). In addition, the WB analysis confirmed these findings (Figure 2G,H). Furthermore, these groups were subjected to immunofluorescence staining for CD206+ and F4/80+ (markers specifically indicative of M2 and M0 macrophages). Immunofluorescence (IF) staining indicated that the DM group had fewer CD206 cells than the NC group, while the NAR + DM group displayed an elevated count (Figure 2I). These observations suggest that naringenin promoted M2 macrophage polarization and decreased inflammation in diabetic wounds.

### 3.3 Naringenin Inhibits Macrophage M1 Polarization While Promoting M2 Polarization

**FIGURE 3:**
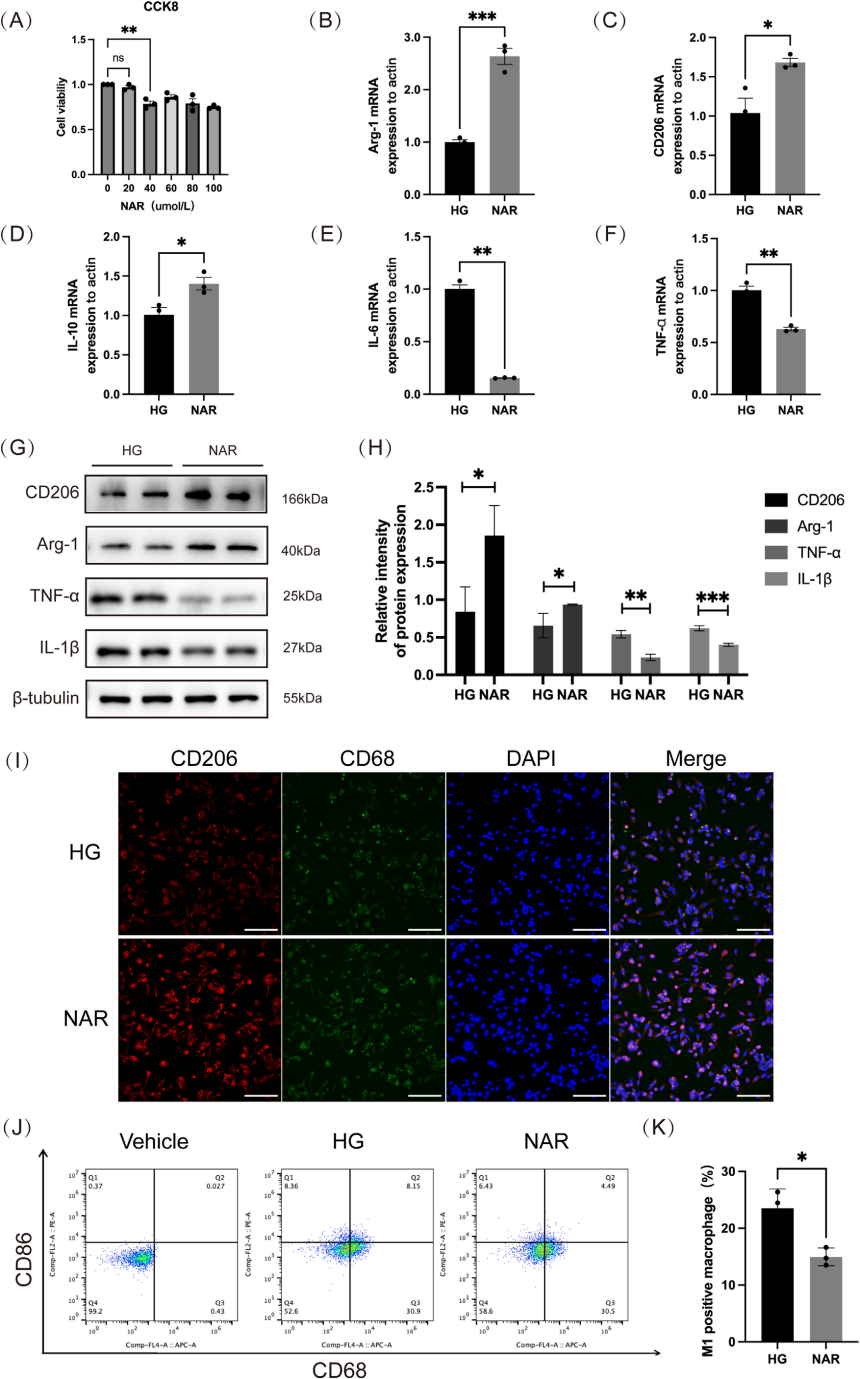
Naringenin promotes macrophages M2 polarization while inhibiting M1 polarization in vitro. (A) A CCK‐8 assay evaluated the impact of varying naringenin concentrations on cell viability. (B–F) mRNA expressions of Arg‐1, CD206, IL‐10, IL‐6, and TNF‐α were detected by qPCR. (G, H) Quantitative analysis of Arg‐1, CD206, IL‐1β, and TNF‐α by western blot in different groups. β‐tubulin served as the internal reference. (I) Immunofluorescence staining showed the CD206^+^ (red) and CD68^+^ (green) cells in different groups; blue fluorescence represents cell nucleus (scale bar, 100 μm). (J, K) Flow cytometry was employed to assess CD86^+^ and CD68^+^ in different groups. The histogram showed quantification for the percentage of M1 macrophages. Data are shown as means ± SEMs (*n* = 3), with significance levels marked as *p < 0.05;**p < 0.01; ***p < 0.001. HG: high glucose medium; NAR: high glucose medium + naringenin.

The CCK‐8 assay evaluated the impact of various naringenin concentrations on THP‐1 cells, revealing that high naringenin levels significantly reduced cell viability. Concentrations above 40 μmol/L decreased viability, while 20 μmol/L showed no significant difference from the control (0 μmol/L). Thus, 20 μmol/L NAR was selected for further experiments (Figure 3A). To assess naringenin's effect on macrophage polarization in a high glucose environment, THP‐1 cells were exposed to LPS in RPMI 1640 medium with 33 mM D‐glucose to mimic chronic diabetes‐induced inflammation.

We investigated the effect of naringenin on THP‐1 cells to determine its potential in promoting macrophage M2 polarization and inhibiting M1 polarization. We initially utilized qPCR to detect the transcription levels of M2 macrophage markers (Arg‐1, CD206, IL‐10) and pro‐inflammatory factors (IL‐6, TNF‐α). The naringenin (NAR) group significantly upregulated CD206, Arg‐1, and IL‐10 expression compared with the high glucose (HG) group, while concurrently diminishing TNF‐α and IL‐6 expression (Figure 3B–F). To further corroborate these findings, we conducted WB assays to verify the expression of M2 macrophage markers (CD206 and Arg‐1) and pro‐inflammatory mediators (TNF‐α, IL‐1β) (Figure 3G,H). Additionally, the IF analysis indicated a notable increase in CD206 expression in the NAR group relative to the HG group (Figure 3I). Moreover, flow cytometry revealed a notable decrease in M1 macrophages (CD86+, CD68+) in the NAR group relative to the HG group (Figure 3J,K). These findings indicate that naringenin facilitates the shift of macrophages to the M2 state and prevents them from transitioning to the M1 state.

### 3.4 Naringenin Decreases the Proportion of M1 Polarization via Activating Nrf2/HO‐1 Signaling Pathway

**FIGURE 4:**
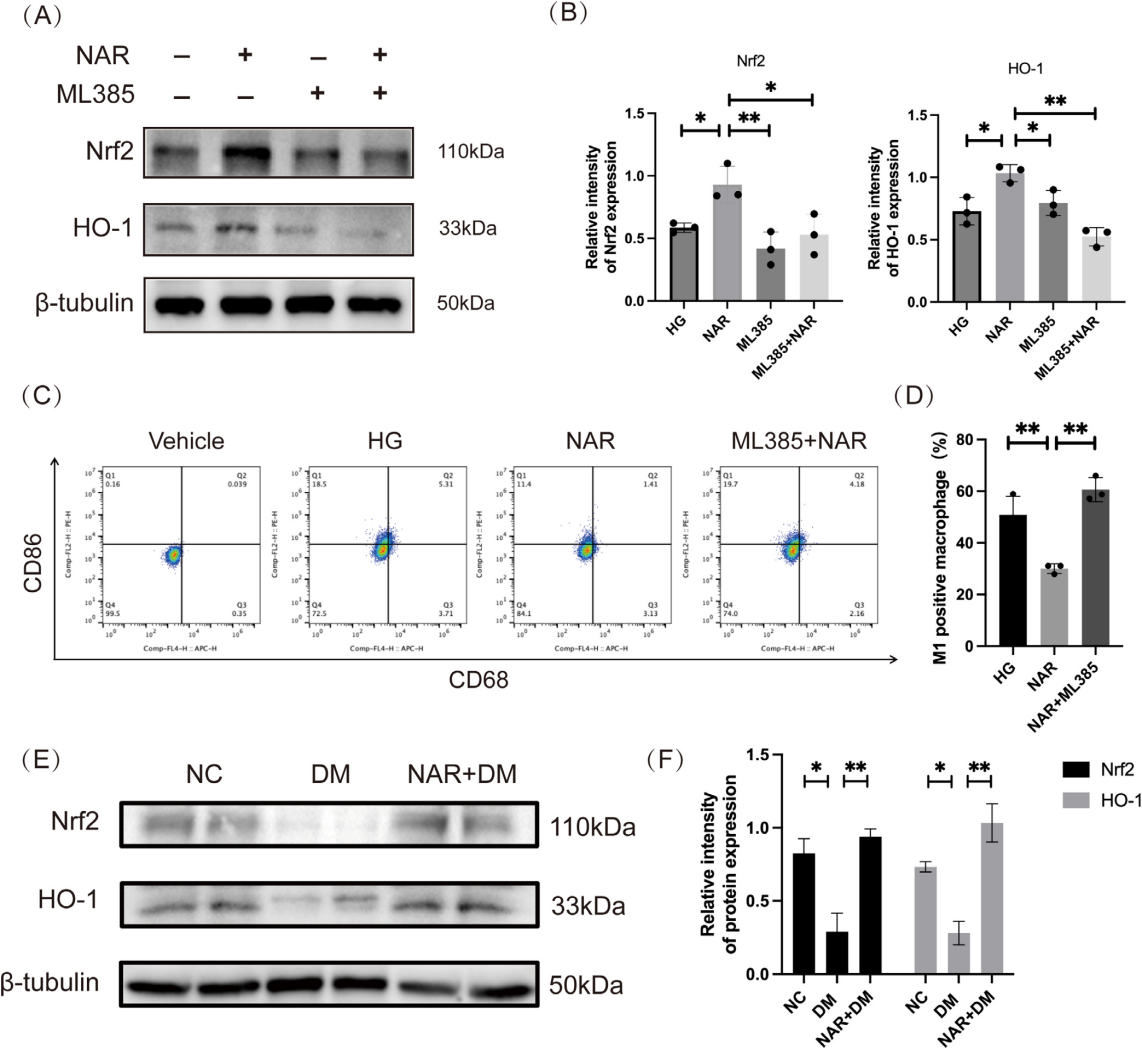
Naringenin inhibits M1 macrophage polarization via Nrf2/HO‐1 signaling pathway. (A, B) The expression levels of Nrf2 and HO‐1 proteins were quantitatively assessed in different groups using western blot. β‐tubulin served as the internal reference. (C) Flow cytometry analysis showed the percentage of M1 macrophage polarization using M1 macrophage markers (CD86+, CD68+). (D) The percentage of CD86+ macrophages is quantified in the histogram. (E, F) Quantitative analysis of the protein expression of Nrf2 and HO‐1 in wound tissues from various groups using western blot. β‐tubulin served as the internal reference. Data are shown as means ± SEMs (*n* = 3), with significance levels marked as *p < 0.05; **p < 0.01; ***p < 0.001. HG: high glucose medium; NAR: high glucose medium + naringenin; ML385: high glucose medium + ML385; NAR + ML385: high glucose medium + naringenin + ML385; NC: group without treatment; DM: group with diabetes; NAR + DM: group with diabetes treated with naringenin.

Prior investigations have indicated that the activation of the Nrf2/HO‐1 pathway has an effect on macrophage polarization (Lv et al. 2021; Sha et al. 2023). Therefore, we focus on the relationship between naringenin and the Nrf2/HO‐1 signaling pathway. In order to clarify naringenin's impact on macrophage polarization, THP‐1 cells were exposed to the Nrf2/HO‐1 pathway inhibitor ML385 for 24 h before naringenin treatment. The WB analysis indicated that naringenin treatment significantly elevates HO‐1 and Nrf2 levels in THP‐1 derived macrophages, and this effect is reversed by ML385 (Figure 4A,B). Flow cytometry results indicated that the NAR + ML385 group had a notably higher proportion of M1 macrophages than the NAR group (Figure 4C,D). We subsequently validated the presence of Nrf2 and HO‐1 in the tissues of the wound. The DM group showed a marked decline in Nrf2 and HO‐1 production relative to the NC group; however, this was reversed by the topical application of naringenin (Figure 4E,F). Given these findings, it can be inferred that naringenin inhibits M1 macrophage polarization through activation of the Nrf2/HO‐1 pathway in diabetic wounds.

### 3.5 Naringenin Promotes Macrophage Efferocytosis In Vitro

**FIGURE 5:**
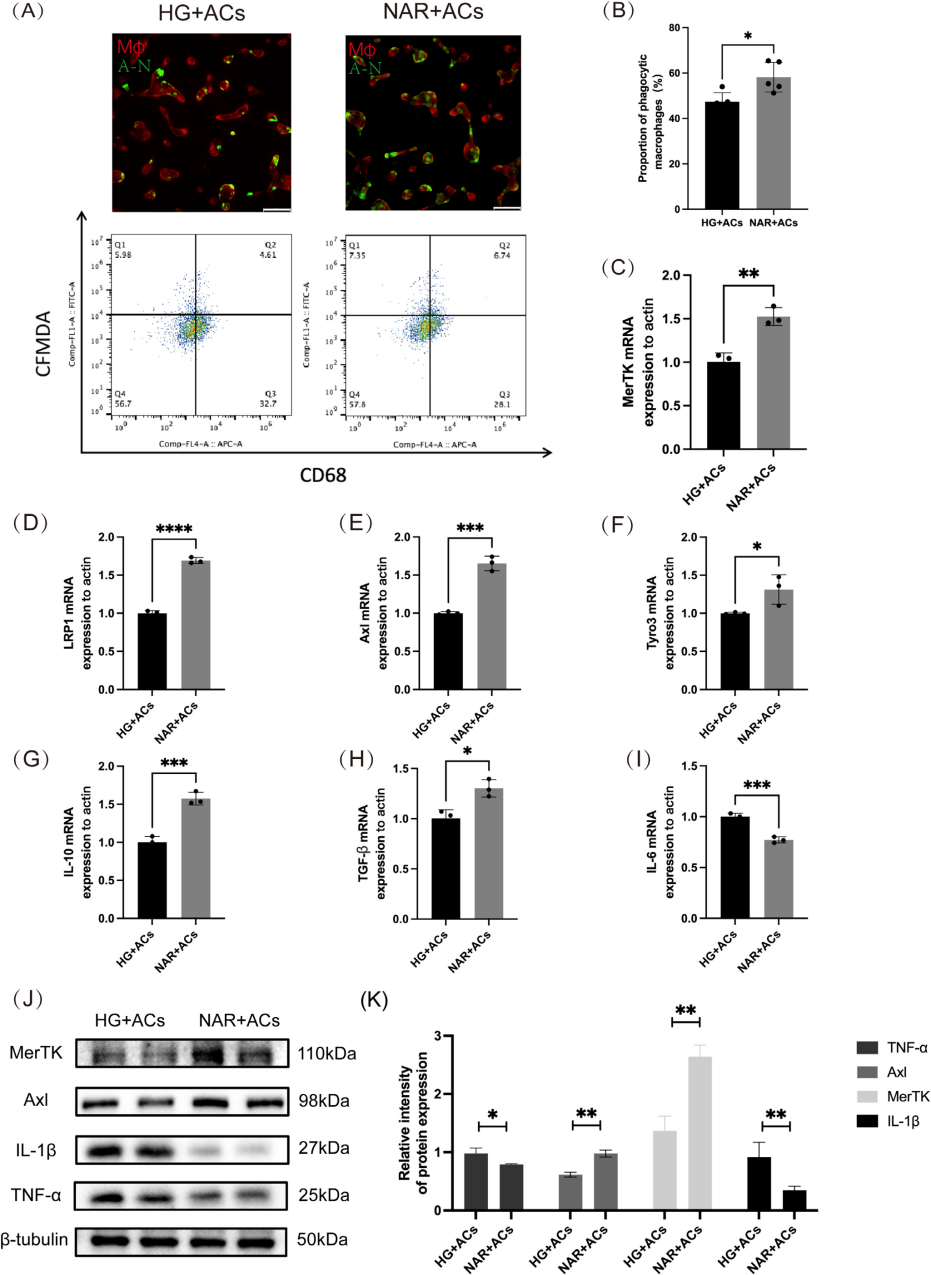
Naringenin promotes macrophages efferocytosis in vitro. (A) Efferocytosis was assessed by immunofluorescence and flow cytometry. Macrophages induced by THP‐1 co‐cultured with CMFDA‐labeled (green) apoptotic Jurkat cells (scale bar, 50 μm). (B) The proportion of phagocytic macrophages was assessed by flow cytometry. (C–I) mRNA expression levels of Axl, MerTK, Tyro3, LRP1, IL‐10, TGF‐β, and IL‐6 were detected by qPCR. (J, K) Quantitative analysis the expression of Axl, MerTK, IL‐1β, and TNF‐α by western blot. β‐tubulin served as the internal reference. Data are shown as means ± SEMs (*n* = 3), with significance levels indicated as *p < 0.05;**p < 0.01; ***p < 0.001. HG + ACs: high glucose medium; NAR + ACs: high glucose medium + naringenin.

Previously, M2 macrophage polarization has been reported to promote efferocytosis (Ariel and Serhan 2012; Liu et al. 2008). Further analysis is conducted on the role of naringenin in macrophage efferocytosis under high glucose conditions (33 mM) by combining apoptotic Jurkat cells with THP‐1 cells. We first employed an immunofluorescence assay to visualize efferocytosis (Figure 5A). CMFDA‐stained Jurkat cells were added to THP‐1 cells. Therefore, the proportion of phagocytic macrophage can be assessed by evaluating the number of CMFDA‐stained Jurkat cells in THP‐1 cells relative to all THP‐1 cells number. Similarly, flow cytometry was used to confirm the immunofluorescence data. As shown by immunofluorescence and flow cytometry, the proportion of phagocytic macrophages in the NAR group increased compared with the HG group (Figure 5B). The expression of genes concerning efferocytosis (Tyro3, MerTK, LRP1, Axl), anti‐inflammatory factors (IL‐10, TGF‐β), and pro‐inflammatory mediators (IL‐6) was evaluated using qPCR (Figure 5C–I). To validate these results, Western Blot analysis was performed to verify the expression of pro‐inflammatory factors (IL‐1β, TNF‐α) and genes related to efferocytosis (Axl, MerTK) (Figure 5J,K). These findings collectively imply that naringenin improves macrophage efferocytosis and diminishes inflammatory responses in vitro.

### 3.6 Naringenin Enhances the Efferocytosis in Diabetic Wound

**FIGURE 6:**
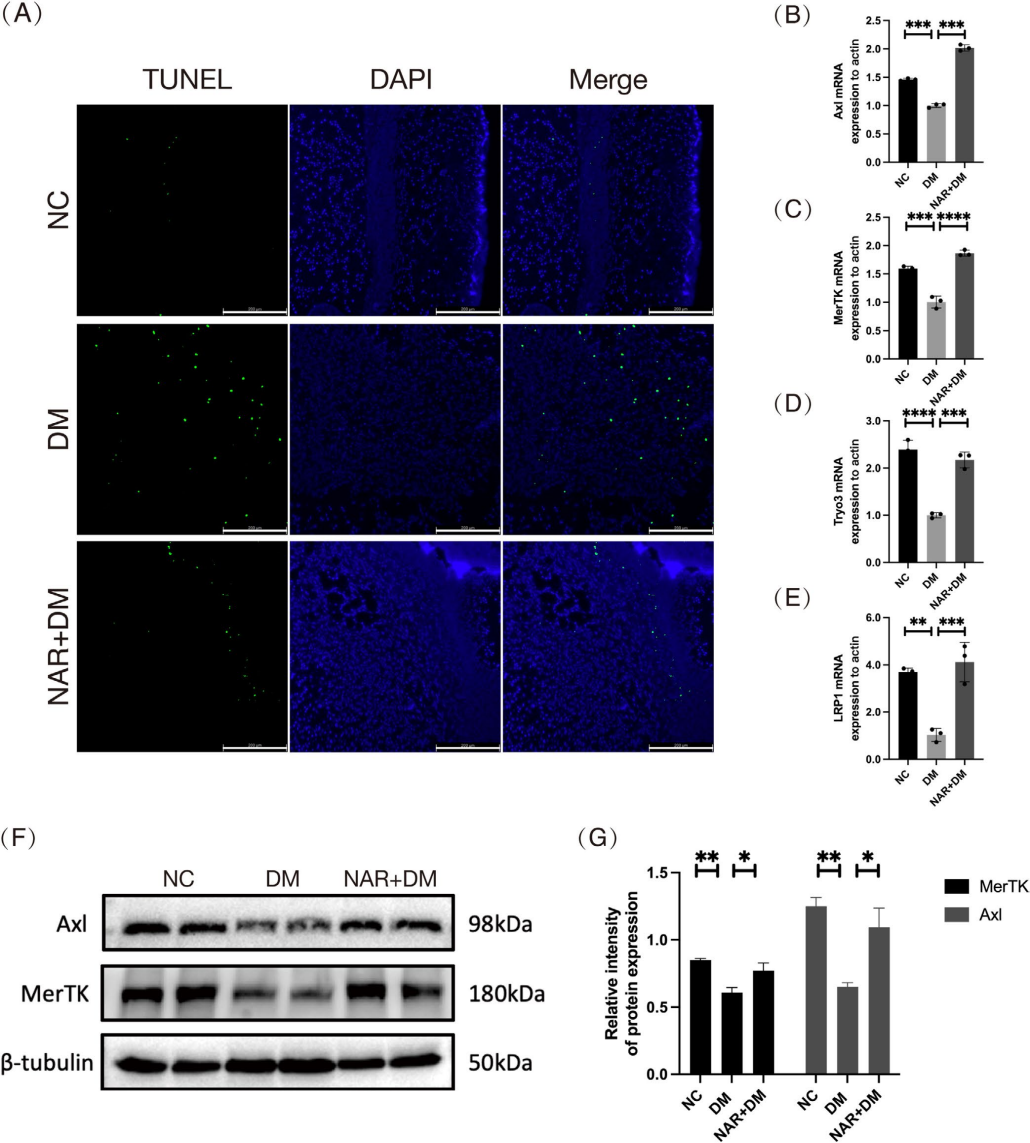
Naringenin promotes macrophage efferocytosis in diabetic wound tissues. (A) TUNEL staining (green: TUNEL; blue: DAPI; scale bar, 200 μm) in different groups. (B–E) mRNA expression levels of Axl, MerTK, Tyro3, LRP1 were detected by qPCR in wound tissues. (F, G) Quantitative analysis of Axl and MerTK expression by western blot from different groups in wound tissues. β‐tubulin served as the internal reference. Data are shown as means ± SEMs (*n* = 3), with significance levels indicated as *p < 0.05; **p < 0.01; ***p < 0.001. NC: group without treatment; DM: group with diabetes; NAR + DM: group with diabetes treated with naringenin.

We then verified the effect of naringenin on efferocytosis in diabetic wounds. We first observed the apoptotic cells in the wound‐edge tissues to evaluate the effect of naringenin on macrophage efferocytosis. TUNEL staining was employed to assess apoptotic cell accumulation in the wound area. As expected, there are fewer green cells in the NC group relative to the DM group. The application of naringenin reduced apoptotic cells (Figure 6A). Subsequently, we assessed efferocytosis‐related genes (MerTK, Axl, Tryo3, LRP1) in the wound areas by qPCR. Compared to the NC group, the DM group exhibited decreased expression of genes associated with efferocytosis, which was improved by naringenin treatment (Figure 6B–E). Moreover, WB analysis supported that the NAR + DM group had elevated efferocytosis marker levels in the wound area relative to the DM group (Figure 6F,G). Thus, naringenin enhances macrophage efferocytosis in diabetic wound healing.

## 4 Discussion

Chronic wounds associated with diabetes are common and are primarily due to sustained inflammation, which impairs the healing process (Zhao et al. 2016). Naringenin, a flavonoid found in nature, is known for its anti‐inflammatory effects and possible therapeutic uses in diabetes‐related issues (Shi et al. 2009; Zhang et al. 2017). In addition, naringenin exhibits distinct characteristics that differentiate it from other flavonoids commonly used in diabetic wound management. Naringenin's application in advanced delivery systems, such as hydrogels, emphasizes its role in restoring immune microenvironments and promoting angiogenesis, which may not be as extensively studied for other flavonoids like quercetin, which is often highlighted for its broad antimicrobial properties in biofilm inhibition (Warrier et al. 2025). Moreover, naringenin modulates multiple pathways, including MMPs and nitric oxide signaling (Chanu et al. 2023), aligning with its potent antioxidant and anti‐inflammatory activities that support macrophage modulation (Uçar and Göktaş 2023). In summary, these unique attributes highlight naringenin's potential as a targeted therapeutic agent in diabetic wound healing, especially in regulating macrophage phenotypes. This study investigates naringenin's impact on diabetic wound healing and elucidates its underlying mechanisms. First, our findings indicate that the topical application of naringenin significantly enhances wound healing in diabetic mice. Second, naringenin has been found to increase macrophage M2 polarization and decrease M1 polarization both in vivo and in vitro. Third, the Nrf2/HO‐1 inhibitor ML385 was found to diminish the inhibitory impact of naringenin on M1 macrophages. Finally, naringenin promotes macrophage efferocytosis both in vitro as well as in vivo. These findings offer new perspectives for treating non‐healing diabetic wounds.

The process of wound healing involves four connected phases: hemostasis, inflammation, proliferation, and tissue remodeling (Rohm et al. 2022). An extended duration of the inflammation stage could potentially impede the subsequent proliferative phase, thus delaying overall wound healing (Deng et al. 2022). Existing research suggests that this sustained inflammation may be due to the unsuccessful M2 macrophage polarization and impaired efferocytosis during the inflammatory stage (Jun et al. 2015; Xie et al. 2022; Zeng et al. 2013). Our research indicates that the healing of wounds in the DM group was considerably slower than that of the NC group. Previous studies indicate that inhibiting inflammation may enhance wound healing (Jiang et al. 2022). Our study shows that naringenin accelerates diabetic wound healing by reducing inflammatory factors, thereby aiding the shift from inflammation to remodeling.

Macrophages, integral components of the innate immune system, are pivotal in the process of wound healing (Munadziroh et al. 2022). Macrophages facilitate wound healing by inducing M2 polarization (Kotwal and Chien 2017; Shapouri‐Moghaddam et al. 2018). In diabetes, hyperglycemia and advanced glycation end products induce the polarization of macrophages into M1 phenotypes, while concurrently inhibiting the formation of M2 macrophages (Jahan and Choudhary 2021). A significant cause of impaired wound healing in diabetes is the uncontrolled increase of pro‐inflammatory M1 macrophages (Wetzler et al. 2000). In diabetic animal models, enhancing wound healing can be achieved by therapies that either stimulate macrophages M2 polarization or reduce M1 polarization (Chen et al. 2015). Furthermore, research indicates that naringenin acts as a positive regulator for M2 macrophage polarization in conditions such as abdominal aortic aneurysm and arthritis (Bussmann et al. 2019; Jia et al. 2022). Prior investigations have demonstrated that naringenin decreases the production of IL‐6 and TNF‐α in colon macrophages, thereby reducing inflammation (Dou et al. 2013). According to our findings, naringenin diminishes pro‐inflammatory factors and M1 macrophage polarization, while encouraging M2 polarization.

Macrophage polarization can be controlled by various signaling pathways (Marrero‐Berrios et al. 2024), with the Nrf2/HO‐1 pathway being particularly significant (Lv et al. 2021; Sha et al. 2023). Evidence from studies indicates that upregulating Nrf2 expression might inhibit the polarization of M1 macrophages and promote the M2 polarization (Kobayashi et al. 2016; Liang et al. 2022; Wang and He 2022). Moreover, evidence indicates that a rise in HO‐1 has a substantial immunomodulatory effect on macrophage polarization (Weis et al. 2009). Our study found that naringenin treatment significantly elevated Nrf2 and HO‐1 levels, but this effect can be counteracted by ML385. The data indicate that naringenin inhibits M1 macrophage polarization via activation of the Nrf2/HO‐1 signaling pathway. Moreover, we found that naringenin increased the levels of HO‐1 and Nrf2 in diabetic wound tissues. These findings propose that naringenin inhibits M1 macrophage polarization in diabetic wounds via the Nrf2/HO‐1 signaling pathway.

Studies conducted previously have shown that M2 macrophage polarization enhances macrophage efferocytosis (Ariel and Serhan 2012; Bystrom et al. 2008). Additionally, current research suggests that dysfunction in macrophage efferocytosis may exacerbate inflammation and impede diabetic wound healing (Kohno et al. 2021). Correcting impaired efferocytosis is gaining recognition as a potential therapeutic approach to address the chronic inflammation in diabetic wounds. Efferocytosis encompasses three distinct processes: recruitment, recognition, and phagocytosis (Lin et al. 2022). During the recognition phase, a variety of receptors located on phagocytes, such as MerTK, Axl, and Tyro3, identify apoptotic signals and regulate inflammation (Cai and Kasikara 2020). The decrease in these indicators suggests that efferocytosis was inhibited. A study found that TNF‐α signaling can reduce MerTK and LRP1 expression, hindering apoptotic cell clearance, exacerbating inflammation, and perpetuating a harmful cycle (Zhang et al. 2022). When co‐culturing THP‐1 cells with apoptotic Jurkat cells, the treatment of naringenin enhanced macrophage efferocytosis and downregulated inflammatory factors. Administering naringenin to the wounds of diabetic mice resulted in a significant reduction in apoptotic cells, along with an increase in efferocytosis markers, specifically MerTK and Axl. These findings suggest that naringenin promotes efferocytosis in diabetic wound tissues. Our study demonstrates that naringenin promotes M2 macrophage polarization and enhances efferocytosis both in vitro and in vivo, effectively mitigating inflammation.

Nevertheless, our study has several limitations. First, the THP‐1 human monocyte cell line is widely employed in vitro to investigate macrophage phenotypes such as M2 polarization and efferocytosis. Its advantages include ease of standardized culture and high reproducibility, facilitating the evaluation of drug effects like those of Naringenin (Huang et al. 2025). However, limitations arise because it, being a monocytic cell line, cannot fully replicate the complexity of the in vivo wound microenvironment (including diabetes‐induced inflammatory dysregulation) or account for the dynamic heterogeneity of macrophages, potentially limiting its predictive value in the real wound healing process (Clayton et al. 2024). Thus, in our study, we leveraged the advantages of THP‐1 cells to preliminarily validate the mechanism of Naringenin in promoting M2 polarization and efferocytosis and complemented this with in vivo models to comprehensively assess the compound's efficacy in diabetic wound healing, thereby addressing the shortcomings of the cell line model. Second, although pharmacological inhibition of the Nrf2/HO‐1 pathway provided supportive evidence for its role, future studies should incorporate siRNA knockdown of Nrf2 to confirm causality. Besides, we only verified the roles of two key proteins, Nrf2 and HO‐1, without in‐depth exploration of upstream and downstream mechanisms (e.g., crosstalk with the NF‐κB pathway). In diabetic wound healing, macrophage polarization involves complex dialogues between multiple signaling pathways. Thus, incomplete validation of upstream and downstream pathways may constrain a holistic understanding of Naringenin's mechanistic network. Future work should employ siRNA knockdown or overexpression experiments to systematically explore how Naringenin regulates these pathway intersections, thereby guiding clinical therapeutic strategies more precisely and bridging the current validation gaps. In addition, further experiments on the dose–effect relationship of Naringenin are needed in the future. Last, the article explores Naringenin's potential mechanism in inhibiting macrophage M1 polarization, while lacking the mechanism of macrophage efferocytosis. More investigation is required to understand the underlying processes.

## 5 Conclusion

In conclusion, our findings indicate that naringenin significantly promoted wound healing in diabetic mice by downregulating the inflammation via enhanced macrophage M2 polarization and efferocytosis. Therefore, we evidenced that naringenin has a potential therapeutic effect on diabetic wounds, and its therapeutic mechanism deserves further study.

## Author Contributions

**Beizhi Wang:** conceptualization (equal), investigation (equal), methodology (equal), writing – original draft (equal). **Yumeng Huang:** data curation (equal), methodology (equal). **Youjun Ding:** funding acquisition (supporting), methodology (equal). **Jingyi Chen:** investigation (equal). **Yutong Chen:** investigation (equal), writing – review and editing (equal). **Hao Zhang:** writing – review and editing (equal). **Qian Tan:** conceptualization (equal), funding acquisition (lead), resources (equal), supervision (equal).

## Ethics Statement

The Animal Ethics Committee of the First Nanjing Hospital approved the animal experiments (approval number: DWSY‐23128728).

## Conflicts of Interest

The authors declare no conflicts of interest.

## Data Availability

The original contributions presented in the study are included in the article. Further inquiries can be directed to the corresponding authors.
